# Spinal cord stimulation for the treatment of painful diabetic neuropathy and risk of major adverse cardiovascular events, mortality, amputation, infection and suicide: a retrospective cohort study

**DOI:** 10.1016/j.eclinm.2025.103489

**Published:** 2025-09-26

**Authors:** Alex E. Henney, Bernhard Frank, David R. Riley, Matthew Anson, Jamie Burgess, Gema Hernadez, Gregory Y.H. Lip, Rayaz A. Malik, Solomon Tesfaye, Daniel J. Cuthbertson, Uazman Alam

**Affiliations:** a Department of Cardiovascular and Metabolic Medicine, University of Liverpool, Liverpool, UK; b Department of Diabetes, Obesity and Endocrinology, University Hospital Aintree, Liverpool University NHS Foundation Trust, Liverpool, UK; c Liverpool Centre for Cardiovascular Science at University of Liverpool, Liverpool John Moores University and Liverpool Heart & Chest Hospital, Liverpool, UK; d Department of Pain Medicine, The Walton Centre, NHS Foundation Trust, Liverpool, UK; e Department of Musculoskeletal and Ageing Science, Institute of Life Course and Medical Sciences, University of Liverpool, Liverpool, UK; f Pain Research Institute, Clinical Sciences Centre, Aintree University Hospital, University of Liverpool, Liverpool, UK; g TriNetX LLC, Cambridge, MA, USA; h Research Division, Weill Cornell Medicine-Qatar, Qatar Foundation, Education City, Qatar; i Diabetes Research Unit, Sheffield Teaching Hospitals NHS Foundation Trust, Sheffield, United Kingdom

**Keywords:** Painful diabetic neuropathy, Spinal cord stimulation, Type 2 diabetes, Cardiovascular disease

## Abstract

**Background:**

Spinal cord stimulation (SCS) has recently been approved by the US Food and Drug Administration (FDA) for the treatment of refractory painful diabetic neuropathy (PDN), although the current evidence for efficacy is limited by small sample sizes and short follow-up. We aimed to assess the benefits of SCS, compared to combination pharmacotherapy (gabapentinoid plus duloxetine), in a large population of PDN patients using real-world evidence.

**Methods:**

We performed a real-world cohort study of electronic medical records using the TriNetX network, a global federated database (9th September 2024). The treatment arm was patients with PDN (defined as diabetes plus neuropathic pain treatment) treated with SCS (refractory to pharmacotherapy), compared against a reference arm of patients treated with gabapentinoid and duloxetine combination therapy. We propensity score matched (1:1) for confounders with 3 years of follow-up. The primary outcome was time-to incident major adverse cardiovascular event (MACE, that is, a composite outcome of non-fatal ischaemic heart disease, cerebrovascular accident or heart failure, or sudden cardiac death) and secondary outcomes included time-to all-cause mortality, below-knee amputation (BKA), suicide (suicidal ideation and/or attempt), staphylococcus aureus infection, major adverse kidney events (end stage renal failure and/or dialysis), diabetes-related ophthalmic disease (retinopathy and/or maculopathy), hospitalisation and SCS explantation. All outcomes were assessed using ICD-10/CPT codes. Stratified analyses assessed the impact of sex, ethnicity, age, geographic location (USA) and discontinuation of analgesia post SCS implantation on these outcomes.

**Findings:**

We identified 145,380 patients, with 3212 treated with SCS and 142,168 treated with dual pharmacotherapy alone. After PSM, 3105 patients were included in each arm. Treatment with SCS significantly reduced the risk of MACE (hazard ratio 0.57 [95% CI 0.49, 0.67]), all-cause mortality (0.49 [0.39, 0.62]), BKA amputation (0.19 [0.08, 0.46]), suicide (0.36 [0.22, 0.59]), staphylococcus aureus infection (0.67 [0.51, 0.90]), MAKE (0.46 [0.27, 0.79]), diabetes-related ophthalmic disease (0.33 [0.23, 0.48]) and hospitalisation (0.59 [0.53, 0.66]), compared with combination pharmacotherapy. 11.1% of SCS patients underwent explant. Female and older patients had greater reductions in BKA and infection with SCS, whilst the associated reduced risk of suicide was most prominent in younger adults.

**Interpretation:**

Our results suggest that SCS treatment of PDN is associated with a reduced risk of MACE, all-cause mortality, major amputation, suicide, and staphylococcus aureus infection. The findings provide evidence for the putative benefits of SCS as treatment for PDN. Prospective sham controlled RCTs with pain endpoint whilst incorporating longer term follow-up of CV/mortality outcomes are needed to confirm the benefits and explore cost-effectiveness of SCS.

**Funding:**

None.

**Research in context**

**Evidence before this study**

We searched PubMed, Embase, and Web of Science. Search terms included “spinal cord stimulation”, “painful diabetic neuropathy”, “nerve fibre pathology”. Reported endpoints included pain scores, neuropathy-specific quality of life measures, nerve conduction parameters, intra-epidermal nerve fibre density on skin biopsy, autonomic function, and survival. Most randomised trials (sample sizes typically <100; follow-up ≤24 months) demonstrated substantial pain relief and functional improvement with SCS compared to best medical therapy. For example, the SENZA-PDN trial (n = 216) reported ≥50% pain reduction in 79% of patients at 6 months versus 5% in the control group, with concurrent improvements in quality of life and sleep. Several smaller mechanistic studies demonstrated increases in intra-epidermal nerve fibre density and improved autonomic function following SCS, though these were of short duration and not powered for cardiovascular or mortality outcomes. Long-term real-world evidence for systemic outcomes such as major adverse cardiovascular events (MACE), amputation, or death in this population was absent.

**Added value of this study**

To our knowledge, this is the first large-scale, propensity score–matched, real-world study to assess the association between SCS use in refractory PDN and long-term systemic outcomes, including cardiovascular events, mortality, and limb amputation. Using a large federated electronic health record network, we found that SCS was associated with substantial reductions in the risk of MACE, all-cause mortality, and below-knee amputation compared with combination pharmacotherapy. The observed hazard ratios for MACE (0.57, 95% CI 0.49–0.67), mortality (0.49, 95% CI 0.39–0.62), and amputation (0.19, 95% CI 0.08–0.46) are of a magnitude comparable to established cardioprotective interventions in high-risk populations. These associations persisted across multiple sensitivity analyses. Our study expands the SCS evidence base beyond neuropathic symptom control, suggesting potential systemic benefits not captured in prior short-term RCTs.

**Implications of all the available evidence**

When integrated with prior RCTs and mechanistic studies showing improvements in pain, functional outcomes, and nerve fibre pathology, our findings suggest that SCS may confer additional long-term benefits in reducing cardiovascular and limb complications and improving survival in refractory PDN. However, given the observational design and possibility of residual confounding, these results should be interpreted with caution. Confirmation in prospective, randomised, or pragmatic trials powered for systemic outcomes is warranted. If validated, these benefits could broaden the clinical indications for SCS, influencing guideline recommendations and encouraging earlier use in selected patients with PDN who remain refractory to pharmacotherapy.

## Introduction

Data from the International Diabetes Federation suggests over 500 million people are living with diabetes globally, projected to rise to ∼800 million people by 2045.^1^^,^^2^ Around 50% will develop diabetic peripheral neuropathy (DPN), and at least 30% painful diabetic neuropathy (PDN).^3^^,^^4^ PDN is associated with neuropathic pain, depression, anxiety, and poor quality of life, and is significantly associated with an increased risk of all-cause mortality, likely through major adverse cardiovascular events (MACE),^5^ as well as an increased suicide risk.^6^ Additionally, severe infections in patients with diabetic foot ulceration increases the risk of below-knee amputation.^7^

First line pharmacotherapy for PDN includes tricyclic antidepressants, serotonin noradrenaline reuptake inhibitors (SNRIs) (i.e., duloxetine), and gabapentinoid-based anticonvulsants (i.e., pregabalin, gabapentin).^8^ The OPTION DM trial demonstrated that first-line drugs and their combinations have comparable efficacy, and maximal tolerated combination treatment provides better relief than maximum tolerated monotherapy.^9^ However, around 50% of patients have refractory and debilitating symptoms despite pharmacotherapy,^10^ whilst others are unable to tolerate medication due to burdensome side effects.^11^ Spinal cord stimulation (SCS) is a recognised treatment option for peripheral neuropathic pain and complex regional pain syndrome^12^ and is an emerging treatment option for PDN. Tesfaye et al. conducted the first trial of SCS in PDN in 1996.^13^ Recently, two devices that deliver SCS have been approved by the US Food and Drug Administration (FDA) and European Medicines Agency (EMA); a high-frequency 10-kHz (HF-SCS) stimulation system that was approved by the FDA in 2021, and a traditional low-frequency (tonic/paraesthesia-based) system that was cleared by the FDA in 2022.^14^ Other devices have since been approved by the FDA. A 2023 international consensus advocated the use of SCS, especially HF-SCS, as a treatment for PDN, not responsive to first- or second-line monotherapy/combination therapy.^15^

To date, the efficacy of SCS has been evaluated in small, but well designed, studies (largest n = 216, SENZA-DPN).^16^ Moreover, studies assessing SCS in patients with PDN are often limited to evaluating pain relief over short follow-up (typically 16–18 months; although there has been recent 24–48-month clinical trial data, as well as 10-year follow-up data from the Maastricht study). SCS has also been linked to an increase in lower limb blood flow and a reduction in amputation rates.^17^ However, the effect of SCS on other clinically meaningful endpoints, such as MACE, mortality, below-knee amputation, staphylococcus aureus infection and suicide, have not been evaluated.

Therefore, the aims of the current study were to assess whether patients with PDN treated with SCS, compared to combination pharmacotherapy (gabapentinoids and duloxetine), would have a lower incidence of MACE, mortality, below-knee amputation, staphylococcus aureus infection and suicide.

## Methods

### Study design and cohort

We conducted a cohort study with anonymised data from TriNetX (TriNetX LLC, Cambridge, MA, USA), a global federated health research network with access to both inpatient and outpatient electronic medical records (EMRs) health care organisations internationally; primarily secondary, and tertiary care providers in North America and Western Europe. This analysis was conducted on the Global Collaborative Network, which contains data from over 135 million patients with access to diagnoses, procedures, medications, laboratory values and genomic information worldwide. The data used in this study was collected on the 9th of September 2024. Further details on the network have been described elsewhere.^18^

**Fig. 1:**
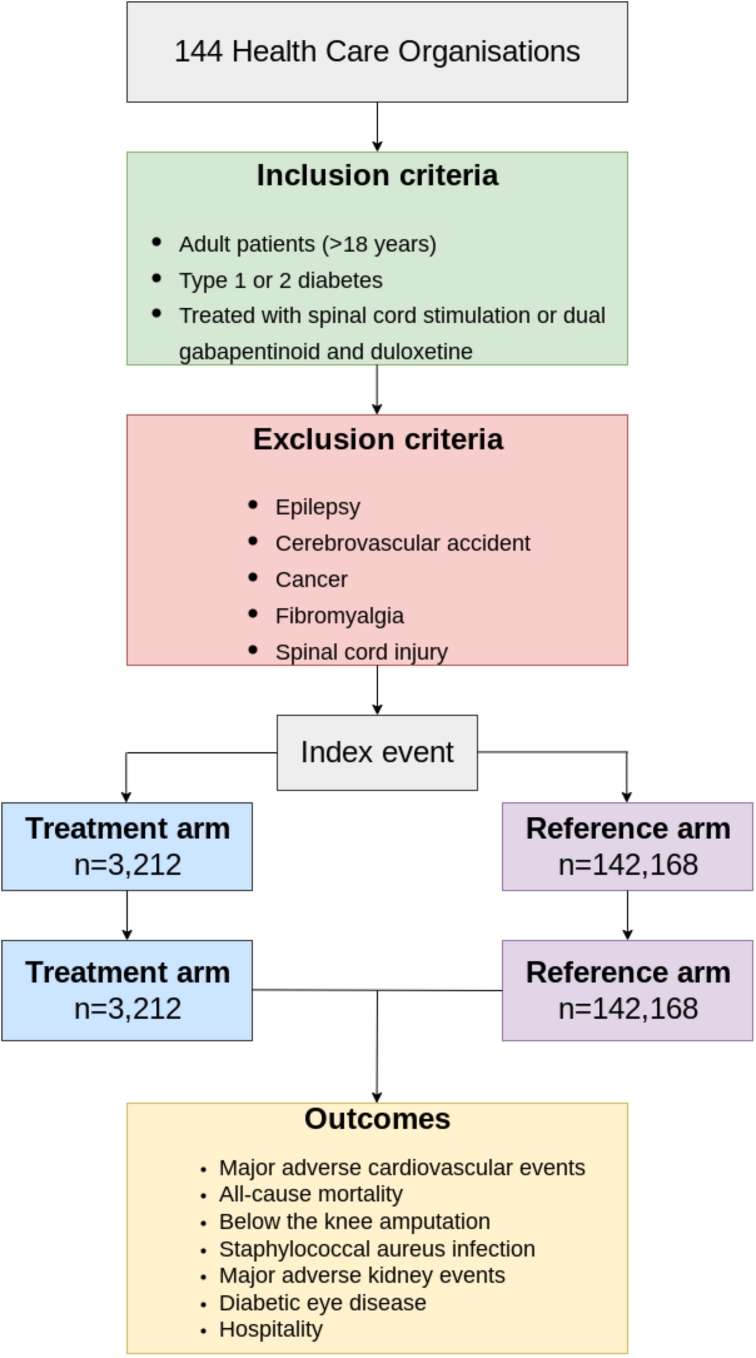
Flow diagram demonstrating the cohort creation process. SCS; spinal cord stimulation.

We identified all patients with PDN (defined as diabetes (either T1D or T2D (diagnosed using one of: **i)** ICD-10 code E10 (T1D) or E11 (T2D); **ii)** HbA1c >6.4%; or **iii)** prescribed glucose-lowering therapy (with the exception of sodium-glucose co-transporter 2 inhibitors (approved for the treatment of heart failure and chronic kidney disease) and the novel glucagon-like peptide-1 receptor agonists (approved for the treatment of obesity)) requiring treatment for neuropathic pain). Two cohorts were subsequently created: **i)** a treatment arm of patients who had received permanent SCS implantation (refractory to combination gabapentinoid and duloxetine therapy), and **ii)** a reference arm of patients who were prescribed combination gabapentinoid and duloxetine therapy without SCS implantation (Fig. 1). SCS was identified using the Current Procedural Terminology (CPT) code 63,685 (for insertion or replacement of a spinal neurostimulator pulse generator or receiver). Treatment and reference arm drugs must have been initiated at least 3 years ago to ensure adequate follow-up and must have occurred after the diagnosis of diabetes. We included an unlimited look-back period throughout their medical history to obtain baseline characteristics. Patients were excluded if they had a diagnosis of any other indication for SCS including epilepsy, cerebrovascular accident, cancer (including chemotherapy-induced peripheral neuropathy), fibromyalgia, or spinal cord injury at any level, prior to the index event. Definitions for inclusion and exclusion criteria are presented in Supplementary Table S1.

### Ethics

We used de-identified, retrospective electronic health record data from the TriNetX Network. TriNetX is compliant with the Health Insurance Portability and Accountability Act (HIPAA), conforms to the General Data Protection Regulation (GDPR), and is certified under the International Organization for Standardization (ISO) standard 27,001 for information security management. Data de-identification adheres to HIPAA standards, specifically Section 164.514(a) for de-identification and 164.514(b)^1^ requiring expert determination. This expert determination was last refreshed in December 2020. Because the data are fully de-identified and collected retrospectively, the study was exempt from institutional ethics committee or institutional review board approval.

### Procedures

**Fig. 2:**
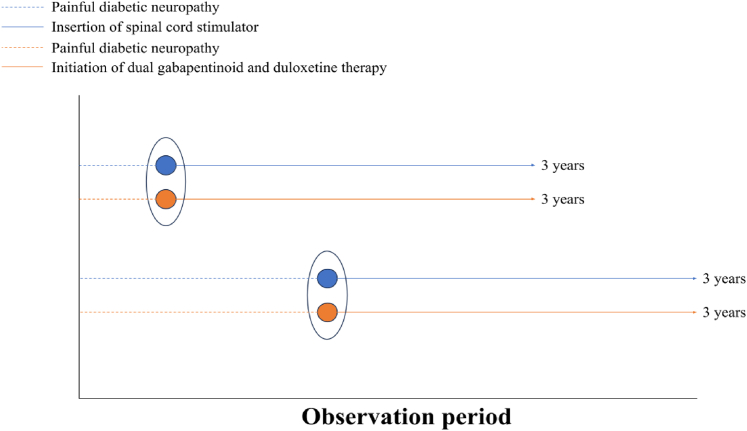
Representation of the active comparator new user design employed in this study.

The index event followed an active comparator, new user design, where analysis was of new users of patients implanted with a SCS device, or started on dual analgesia, one day after treatment initiation. For dual analgesia, the index event took the date of which the latter most drug was initiated. Patients were followed up for 3 years (Fig. 2). The active comparator design serves to reduce time-related bias and provides results more applicable to clinical practice considering the reference/comparator (in this case gabapentinoid and duloxetine combination therapy) is an alternative treatment option for more refractory PDN, enhancing the generalisability of our findings to broader patient populations.

Cohorts were propensity score matched (PSM), in a 1:1 ratio, for: **i) demographics** (age, sex, ethnicity, smoking, alcohol-use disorder, cocaine use, socioeconomic status (problems relating to education and literacy, employment, housing, and psychosocial circumstances), **ii) anthropometrics** (body mass index), **iii) biochemistry** (HbA1c, estimated glomerular filtration rate (eGFR), vitamin B_12_), **iv) cardiovascular risk factors** (hypertension, and dyslipidaemia), **v) comorbidities** (cardiovascular disease (ischaemic heart disease (IHD), peripheral vascular disease (PVD), heart failure), disorders of vitamin and mineral deficiencies (including Vitamin D deficiency), sleep disorders, migraine, traumatic brain injury (TBI), depression, other peripheral neuropathies, and movement disorders (including Parkinson's Disease)), **vi) medication** (analgesic burden (opioid analgesia, non-steroidal anti-inflammatories (NSAIDs), benzodiazepines, carbamazepine, topiramate, bupropion, anti-depressants (including tricyclic antidepressants, selective serotonin reuptake inhibitors (SSRIs), serotonin–norepinephrine reuptake inhibitor (SNRIs), trazodone, mirtazapine, anti-psychotics, corticosteroids, blood glucose lowering therapies (insulin, metformin, sulfonylureas, thiazolidinediones, glucagon-like peptide-1 receptor agonists, sodium-glucose co-transporter-2 inhibitors, dipeptidyl peptidase-4 inhibitors). Definitions for all PSM covariates are presented in Supplementary Table S2.

**Outcomes** The primary outcome of interest was time-to-incident composite four-point MACE (non-fatal IHD, non-fatal cerebrovascular accident, heart failure, sudden cardiac death). The secondary outcomes of interest were time-to-incident all-cause mortality, suicide (suicidal ideation or attempt), below-knee amputation, staphylococcus aureus infection, major adverse kidney events (MAKE), diabetes-related ophthalmic disease, which encompasses retinopathy and maculopathy, hospitalisation and SCS explantation. Secondary outcomes were chosen because of associations with PDN; for example, pain severity in PDN is associated with levels of anxiety and depression, whilst incidence of staphylococcal aureus infection are also higher in this population.^19^^,^^20^ All definitions used in the identification of outcomes are presented in Supplementary Table S3. Both arms were followed up until the first coding of the respective outcome of interest on their EMRs. Patients who did not develop the respective incident outcome of interest were censored at the: **i)** end of the time window for analysis, or **ii)** patient's last known fact date.

**Statistical analysis** Statistical analysis for cohort data was performed in situ within the ‘Compare Outcomes’ analytical model of the TriNetX platform. Normally distributed baseline characteristics are presented as mean and standard deviation. PSM was performed using logistic regression. TriNetX uses ‘greedy nearest-neighbour matching’ with a caliper of 0.1 pooled standard deviations and difference between propensity scores <0.1. We assessed covariate balance between groups using the standardised mean difference (SMD). SMD <0.1 was considered well matched. The reference arm was considered the reference cohort (hazard ratio = 1) when compared against the treatment arm. Kaplan Meier survival analysis was performed to estimate the probability of an outcome, at daily time intervals, over 3 years from the index event. A hazard ratio, log rank test and Kaplan–Meier survival curve were generated, and a proportionality test to validate the proportional hazards assumption was performed. TriNetX uses the R Survival package version 3.2-3. There is a cox regression running in parallel with the Kaplan Meier survival analysis to calculate the hazard ratio. The difference is that the ‘Compare Outcomes’ TriNetX model is looking at the effect of just the index event on the outcome. This model in effect assumes all other aspect of the cohort are equal, which is why we propensity score match for possible confounders. While the user interface may not explicitly display a stratified Cox model or separate adjustment for the matched pairs, the PSM is performed prior to outcome comparison to balance baseline covariates between groups. This design ensures that the estimated hazard ratio compares well-balanced cohorts, effectively controlling for confounding through matching. Unlike traditional Cox regression models which adjust for multiple covariates simultaneously, the TriNetX approach estimates the hazard ratio for the index event under the assumption that matched cohorts are comparable on measured confounders. Further, we performed stratified analyses by age, sex, ethnicity, specific geographical location (USA), and discontinuation of gabapentinoid-duloxetine therapy after implantation of SCS, whilst we also calculated E-values, representing the minimum strength of association on the HR scale that an unmeasured confounder would need to have with both the exposure (treatment arm) and the outcome, conditional on the measured confounders, to explain away the observed association; HR+√[HR × (HR-1)].^21^ Finally, we assessed rates of SCS explantation. For clarity, the stratified analyses were performed by re-running the analysis with the stratification removed from the propensity score model; for example, when stratified by sex, patients were not matched for sex. The Strengthening the Reporting of Observational Studies in Epidemiology (STROBE) guidelines were followed in the reporting of this cohort study.^22^

### Role of funding source

No funding received.

## Results

We identified a total of 145,380 patients. 3212 (2.2%) were treated with SCS, whilst 142,168 (97.8%) were treated with combination gabapentinoid and duloxetine therapy. On average, patients treated with SCS were older, more likely to be of white ethnicity, and to be prescribed opioid-based analgesia, benzodiazepines, tricyclic antidepressants, antibiotics, corticosteroids, and insulin. Equally, they were less likely to prescribed NSAIDs, non-insulin glucose-lowering therapy (specifically sulphonylureas), or be living with adverse socioeconomic hazards, alcohol-use disorder, T1D, depression, PVD, TBI or vitamin/mineral deficiencies, but had better glycaemic control. After PSM, the cohort was deemed well matched and included 3105 patients in each arm (Table 1).

**Table 1:** Baseline characteristics, pre- and post-matching, for patients treated with spinal cord stimulation and pharmacotherapy. SMD, standardised mean difference.

Variable	Pre-matching			Post-matching		
	Treatment	Reference	SMD	Treatment	Reference	SMD
Patients, n	3212	142,168		3105	3105	
**Demographics**						
Age, years	61 ± 12	59 ± 14	0.15	61 ± 12	61 ± 13	0.02
Female, %	1420 (44.2)	84,021 (59.1)	0.30	1395 (44.3)	1424 (45.2)	0.02
***Ethnicity, n (%)***						
White	2277 (70.9)	91,698 (64.5)	0.14	2230 (70.8)	2237 (71.0)	<0.01
Black	234 (7.3)	20,899 (14.7)	0.24	230 (7.3)	243 (7.7)	0.01
Asian	39 (1.2)	1706 (1.2)	<0.01	38 (1.2)	28 (0.9)	0.03
Hispanic	141 (4.4)	11,942 (8.4)	0.16	139 (4.4)	139 (4.4)	<0.01
Socioeconomic risk factors	35 (1.1)	4692 (3.3)	0.15	35 (1.1)	36 (1.2)	<0.01
**Anthropometrics**						
Body mass index, kg/m^2^	33.6 ± 6.9	34.7 ± 8.6	0.01	33.6 ± 6.9	33.7 ± 7.5	0.02
**Biochemistry**						
eGFR, mL/min/1.73 m^2^	79 ± 23	79 ± 30	0.02	79 ± 23	80 ± 25	0.02
HbA1c, %	7.0 ± 2.1	7.7 ± 2.4	0.31	7.0 ± 2.1	7.1 ± 2.0	0.05
Triglycerides, mg/dL	182 ± 186	181 ± 199	0.01	182 ± 186	181 ± 180	<0.01
HDL cholesterol, mg/dL	43.6 ± 20.8	43.6 ± 18.0	<0.01	43.6 ± 20.8	42.4 ± 17.2	0.06
B12	625 ± 368	630 ± 513	0.01	625 ± 368	593 ± 324	0.09
**Cardiovascular risk factors, n (%)**						
Hypertension	1776 (55.3)	78,477 (55.2)	<0.01	1739 (55.2)	1833 (58.2)	0.06
Diastolic blood pressure	75 ± 11	73 ± 14		75 ± 11	73 ± 14	
Systolic blood pressure	131 ± 18	128 ± 22		131 ± 18	127 ± 21	
Dyslipidaemia	1275 (39.7)	62,412 (43.9)	0.09	1254 (39.8)	1279 (40.6)	0.02
Smoking	488 (15.2)	22,747 (16.0)	0.02	476 (15.1)	504 (16.0)	0.02
Alcohol-use disorder	84 (2.6)	6682 (4.7)	0.11	82 (2.6)	76 (2.4)	0.01
Opiate-use disorder	151 (4.7)	5545 (3.9)	0.04	148 (4.7)	148 (4.7)	<0.01
**Comorbidities, n (%)**						
***Cardiovascular disease***						
Ischaemic heart disease	572 (17.8)	29,002 (20.4)	0.07	561 (17.8)	586 (18.6)	0.02
Peripheral vascular disease	3.8	10,805 (7.6)	0.16	120 (3.8)	129 (4.1)	0.01
***Mental health disorders***						
Sleep disorders	122 (25.7)	36,395 (25.6)	<0.01	810 (25.7)	567 (27.2)	0.03
Depression	726 (22.6)	39,096 (27.5)	0.11	715 (22.7)	753 (23.9)	0.03
***Neurology and neurosurgery***						
Migraine	215 (6.7)	9099 (6.4)	0.01	211 (6.7)	227 (7.2)	0.02
Parkinson's disease	42 (1.3)	1280 (0.9)	0.04	41 (1.3)	39 (1.2)	<0.01
Traumatic brain injury	183 (5.7)	12,795 (9.0)	0.13	180 (5.7)	187 (5.9)	0.01
***Vitamin and mineral metabolism***						
Vitamin D deficiency	241 (7.5)	18,055 (12.7)	0.18	236 (7.5)	255 (8.1)	0.02
Other vitamin and mineral deficiencies	116 (3.6)	11,780 (8.3)	0.20	113 (3.6)	116 (3.8)	0.01
**Medication, n (%)**						
***Analgesia***						
Opioids	2423 (75.6)	90,988 (64.0)	0.26	2378 (75.5)	2460 (78.1)	0.06
NSAIDs	540 (16.8)	30,424 (21.4)	0.12	529 (16.8)	520 (16.5)	0.01
Topiramate	215 (6.7)	6682 (4.7)	0.08	208 (6.6)	217 (6.9)	0.01
Carbamazepine	22 (0.7)	1422 (1.0)	0.03	22 (0.7)	22 (0.7)	<0.01
***Antidepressants***						
Tricyclics	504 (15.7)	17,202 (12.1)	0.11	495 (15.7)	498 (15.8)	<0.01
Trazodone	411 (12.8)	19,335 (13.6)	0.02	406 (12.9)	428 (13.6)	0.02
Venlafaxine	193 (6.0)	7108 (5.0)	0.05	189 (6.0)	208 (6.6)	0.02
Paroxetine	87 (2.7)	3981 (2.8)	0.01	85 (2.7)	91 (2.9)	0.01
Desvenlafaxine	26 (0.8)	853 (0.6)	0.03	25 (0.8)	25 (0.8)	<0.01
Bupropion	270 (8.4)	11,658 (8.2)	0.01	265 (8.4)	292 (9.3)	0.03
Sertraline	196 (6.1)	12,226 (8.6)	0.10	192 (6.1)	195 (6.2)	<0.01
Escitalopram	202 (6.3)	9667 (6.8)	0.02	198 (6.3)	221 (7.0)	0.03
Citalopram	206 (6.4)	9667 (6.8)	0.01	202 (6.4)	205 (6.5)	<0.01
Fluoxetine	154 (4.8)	7677 (5.4)	0.03	148 (4.7)	142 (4.5)	0.01
Mirtazapine	96 (3.0)	5971 (4.2)	0.06	95 (3.0)	101 (3.2)	0.01
Antipsychotics	385 (12.0)	20,899 (14.7)	0.08	378 (12.0)	381 (12.1)	<0.01
Antimigraines	145 (4.5)	5687 (4.0)	0.03	142 (4.5)	154 (4.9)	0.02
Benzodiazepines	1860 (57.9)	63,833 (44.9)	0.26	1821 (57.8)	1859 (59.0)	0.02
Antimicrobials	2117 (65.9)	85,016 (59.8)	0.13	2073 (65.8)	2123 (67.4)	0.03
Corticosteroids	1657 (51.6)	65,682 (46.2)	0.11	1622 (51.5)	1673 (53.1)	0.03
***Glucose-lowering therapy***						
Metformin	1156 (36.0)	65,446 (39.0)	0.06	1134 (36.0)	1225 (38.9)	0.06
Insulin	1153 (35.9)	69,947 (49.2)	0.27	1131 (35.9)	1181 (37.5)	0.03
Glipizide	225 (7.0)	14,928 (10.5)	0.12	224 (7.1)	240 (7.6)	0.02
Glimepiride	206 (6.4)	8530 (6.0)	0.02	202 (6.4)	208 (6.5)	0.01
Glyburide	64 (2.0)	5260 (3.7)	0.10	63 (2.0)	63 (2.0)	<0.01
Linagliptin	48 (1.5)	2843 (2.0)	0.04	47 (1.5)	66 (2.1)	0.04
Sitagliptin	206 (6.4)	11,516 (8.1)	0.06	202 (6.4)	205 (6.5)	<0.01
Liraglutide	132 (4.1)	5545 (3.9)	0.01	129 (4.1)	132 (4.2)	<0.01
Dulaglutide	83.5 (2.6)	4123 (2.9)	0.02	82 (2.6)	88 (2.8)	0.02
Semaglutide	38.5 (1.2)	1422 (1.0)	0.02	38 (1.2)	44 (1.4)	0.02
Tirzepatide	10 (0.3)	142 (<0.1)	0.08	9.5 (0.3)	9.5 (0.3)	<0.01
Empagliflozin	87 (2.7)	3554 (2.5)	0.01	82 (2.6)	79 (2.5)	0.01
Dapagliflozin	26 (0.8)	1706 (1.2)	0.04	25 (0.8)	32 (1.0)	0.02
Canagliflozin	48 (1.5)	2843 (2.0)	0.04	44 (1.4)	57 (1.8)	0.03
Pioglitazone	112 (3.3)	6824 (4.8)	0.08	104 (3.3)	120 (3.8)	0.03

**Fig. 3:**
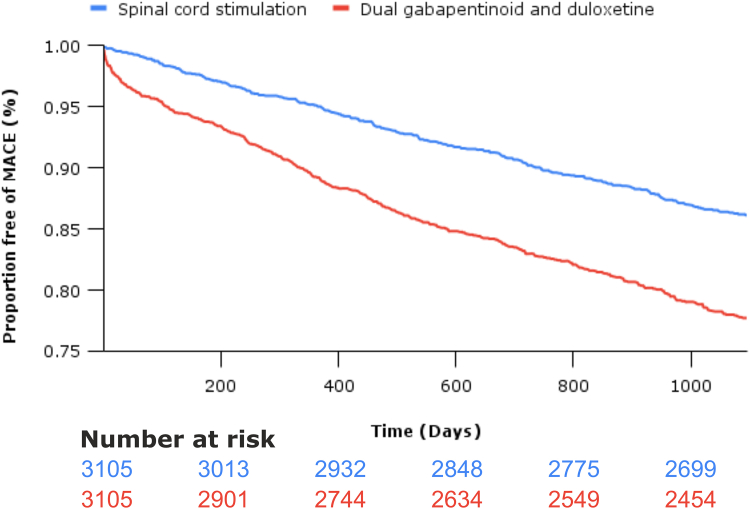
Kaplan Meier survival analysis demonstrating the proportion of patients free of major adverse cardiovascular events (MACE) over 3 years in patients treated with spinal cord stimulation or combination gabapentinoid and duloxetine therapy.

**Fig. 4:**
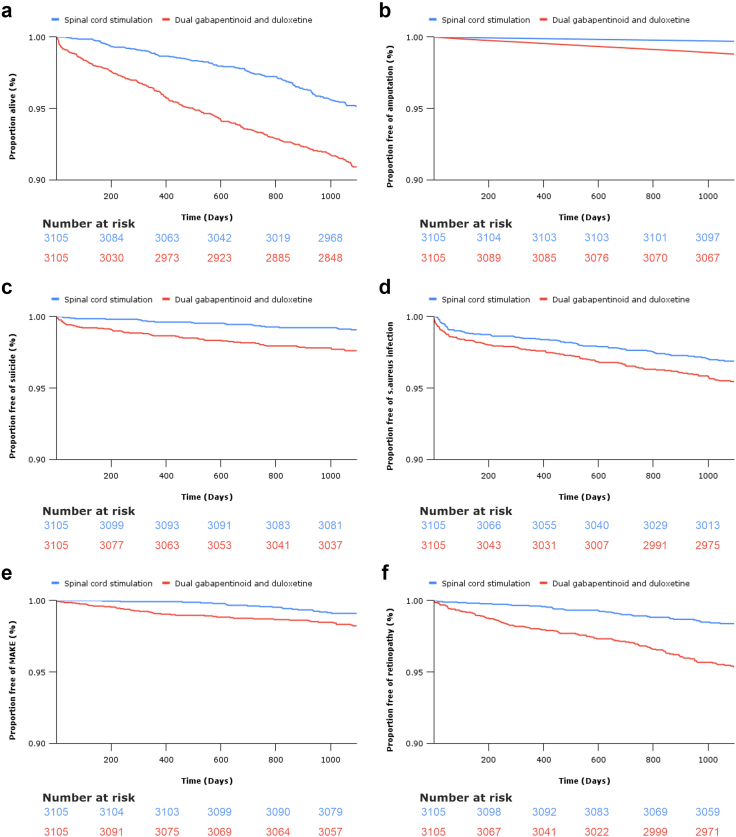
Kaplan Meier **s**urvival analysis demonstrating the proportion of patients free of **a)** all-cause mortality, **b)** below-knee amputation, **c)** suicide, **d)** staphylococcus aureus infection, **e)** major adverse kidney events (MAKE), and **f)** retinopathy, over 3 years in patients treated with spinal cord stimulation or combination gabapentinoid and duloxetine therapy.

Treatment with SCS is associated with significantly reduced risk of the primary outcome, incident MACE (hazard ratio 0.57 [95% CI 0.49, 0.67], p < 0.0001, E-value = 2.91; Fig. 3). Moreover, treatment with SCS significantly reduced the risk of all secondary outcomes (Fig. 4): **i)** all-cause mortality (0.49 [95% CI 0.39, 0.62], p < 0.0001, E-value = 3.50), **ii)** below-knee amputation (0.19 [95% CI 0.08, 0.46], p < 0.0001 E-value = 10.00), **iii)** suicide (0.36 [95% CI 0.22, 0.59], p < 0.0001, E-value = 5.00), **iv)** staphylococcus aureus infection (0.67 [95% CI 0.51, 0.90], p 0.0064, E-value = 2.35), **v)** MAKE (0.46 [95% CI 0.27, 0.79], p 0.0034, E-value 3.77), **vi)** ophthalmic disease (0.33 [95% CI 0.23, 0.48], p < 0.0001, E-value 5.51), and **vii)** hospitalisation (0.59 [95% CI 0.53, 0.66], p < 0.0001, E-value 2.78; Table 2). 345 patients underwent SCS explant (11.1%). The median follow up was 1095 days (interquartile range 479) in the SCS arm and 1095 days (415) in the pharmacotherapy arm.

**Table 2:** Results following survival analysis for the primary and secondary outcomes. Survival probability represents the ‘Survival Probability at End of Time Window’.

	Sample size	Outcome	Survival probability	Hazard ratio [95% confidence interval]	Log Rank	P value	E-value for point estimate [for upper confidence limit]
**Major adverse cardiovascular events**							
Dual gabapentinoid and duloxetine	2255	418	77.7	1.00 [reference]			
SCS	2365	252	86.1	0.57 [0.49, 0.67]	51.3	<0.0001	2.91 [2.34]
**All-cause mortality**							
Dual gabapentinoid and duloxetine	3105	237	90.9	1.00 [reference]			
SCS	3105	114	95.2	0.49 [0.39, 0.62]	40.2	<0.0001	3.50 [2.60]
**Below knee amputation**							
Dual gabapentinoid and duloxetine	3076	32	98.8	1.00 [reference]			
SCS	3095	<10	99.7	0.19 [0.08, 0.46]	17.3	<0.0001	10.0 [3.76]
**Suicidal ideation or attempt**							
Dual gabapentinoid and duloxetine	3105	62	97.6	1.00 [reference]			
SCS	3105	20	99.1	0.36 [0.22, 0.59]	18.3	<0.0001	5.00 [2.77]
***Staphylococcus aureus* infection**							
Dual gabapentinoid and duloxetine	3105	118	95.5	1.00 [reference]			
SCS	3105	78	96.9	0.67 [0.51, 0.90]	7.5	0.0064	2.35 [1.46]
**Major adverse kidney events**							
Dual gabapentinoid and duloxetine	3071	44	98.2	1.00 [reference]			
SCS	3084	20	99.1	0.46 [0.27, 0.79]	8.6	0.0034	3.77 [1.86]
**Retinopathy**							
Dual gabapentinoid and duloxetine	2996	111	95.4	1.00 [reference]			
SCS	3039	37	98.4	0.33 [0.23, 0.48]	37.1	<0.0001	5.51 [3.58]
**Hospitalisation**							
Dual gabapentinoid and duloxetine	3105	845	69.3	1.00 [reference]			
SCS	3105	525	79.2	0.59 [0.53, 0.66]	93.5	<0.0001	2.78 [2.41]

The primary outcome of MACE remained significant after all stratified analyses. The secondary outcomes of all-cause mortality and hospitalisation also remained significant after all stratified analyses. Males (0.27 [95% CI 0.09, 0.80]) receiving SCS were less likely to require below-knee amputations than females (0.34 [95% CI 0.07, 1.69]), however females (0.54 [95% CI 0.34, 0.88]) receiving SCS were less likely to develop staphylococcus aureus infections than males (0.94 [95% CI 0.64, 1.39]). Patients of white ethnicity (0.30 [95% CI 0.18, 0.52], 0.62 [95% CI 0.45, 0.87], and 0.43 [95% CI 0.28, 0.67], respectively) receiving SCS were less likely to have suicidal ideation, staphylococcus aureus infection, and diabetes-related ophthalmic disease, than patients of non-white ethnicity (0.35 [95% CI 0.11, 1.09], 0.83 [95% CI 0.47, 1.47], and 0.56 [95% CI 0.28, 1.14] respectively). Older adults (0.50 [95% CI 0.36, 0.69]) receiving SCS were less likely to develop staphylococcus aureus infection than younger adults (0.94 [95% CI 0.58, 1.51]; Supplementary Table S4).

## Discussion

In this largest study assessing long-term outcomes of SCS in 6074 propensity score matched patients with PDN, using a large real-world data eco-system, we demonstrate that SCS treatment, compared to combination pharmacotherapy (gabapentinoid and duloxetine), was significantly associated with lower risk of MACE (43%), all-cause mortality (51%), suicidal ideation/attempt (64%), below-knee amputations (81%), staphylococcus aureus infection (33%), MAKE (54%), diabetes-related ophthalmic disease (67%), and hospitalisation (41%). These findings have major implications for the treatment of patients with PDN.

To date, eleven clinical trials, four of which were randomised controlled trials (RCTs), demonstrate the effectiveness of both (traditional) low- and high-frequency SCS, compared with best medical treatment, in significantly improving pain in PDN. Burst SCS also appears effective in PDN in a more limited number of trials.^16^^,^^23^^,^^24^^,^^25^ RCTs using low frequency SCS have been limited by an inability to blind participants given that stimulation leads to paraesthesia over the targeted area, resulting in important placebo and nocebo bias. Studies using low frequency SCS have been small and demonstrated a relatively low, but meaningful, effect size.^23^, ^24^, ^25^ Currently, only one RCT (SENZA-PDN) comparing HF-SCS with conventional medical management^16^ has shown greater efficacy in reducing pain for at least 24 months.^26^, ^27^, ^28^ These results were validated in a small real-world evidence retrospective study of 89 PDN patients^29^ which showed that high frequency SCS reduced subjective pain sensation by approximately 80% in at least half of patients, far superior to the 30% reduction in pain in only 30% of patients treated with pharmacotherapy.^30^ The improvements in pain are likely driven by improvements in small nerve fibre pathology, as evidenced by increased proximal and distal intra-epidermal, and corneal, nerve fibre densities over 12 months of treatment.^31^

To the best of our knowledge, this is the largest real-world study to assess the impact of SCS on other (non-pain related) key long-term health outcomes associated with PDN; namely MACE, amputation, suicide and staphylococcus aureus infection. Our findings align with the existing literature demonstrating improved cardiovascular health in patients treated with SCS for other indications.^32^ Thus, 132 patients with IHD treated with SCS (for any indication) experienced significant improvement in quality of life, reduction in angina frequency, and less use of short-acting nitrates.^33^ A recent systematic review of 59 peer-reviewed publications suggests that the improvement in cardiovascular health may be related to cardiovascular autonomic neuromodulation, particularly improved blood pressure,^32^ through a reduction in sympathetic overactivity. Preliminary data suggests that autonomic re-innervation is possible following SCS treatment through improvements in sudomotor axon reflex tests.^34^ It is likely that this may, in part, be related to improved sleep quality with adequately controlled pain.^35^

Reduction in staphylococcal diabetic foot infections may be related to an improvement in sensory profiles in patients treated with SCS^31^; although it is worth highlighting that infection is treated earlier in patients with SCS given the risks associated with an implantable device. We do demonstrate comparable SCS explant rates to existing literature.^36^ Recently, post-hoc analysis of SENZA-PDN demonstrated long-term, significant, and clinically meaningful reductions in HbA1c and body weight, likely related to a reduction in inflammation and metabolic disturbance associated with chronic pain, as well as increased activity after pain relief.^37^ This may further contribute to the reductions in MACE, below-knee amputations and infection seen in the current study. Indeed, in a recent study of patients with foot ulceration and nonhealing wounds due to severe lower limb ischemia, SCS, compared to traditional debridement, showed a significant increase in dorsal foot transcutaneous oxygen tension, and ankle brachial index, as well as reduction in amputation at 6 and 12 months.^38^ Furthermore, in a retrospective observational study, patients with a diabetic foot ulcer in the SCS treatment group had a higher quality-of-life score, larger reduction in pain scores and foot temperature and increase in lower limb vasodilation and nerve conduction velocity compared to endovascular revascularization at six months.^39^

Finally, treatment with SCS reduced suicidal ideation and attempts which may be due to a reduction in pain related anxiety and depression,^19^ as well as the supratentorial effects of SCS.^36^ These findings align with SCS treatment for other indications whereby reduction in anxiety and depression symptoms have been demonstrated.^40^ Crucially, we find that this protective effect against suicide is more pronounced in patients who have SCS treatment earlier in life, which carries clinical significance given that the peak age of suicide (for men) is between the age of 20–40 years.^41^ Together, the results of our study validate the existing literature and add novel findings, whilst having the benefit of being a large-scale real-world study.

Following the CHecklist for statistical Assessment of Medical Papers (CHAMP) guidelines, the hazard ratio estimates with 95% CIs for our primary and secondary outcomes indicate that the observed associations are both statistically robust and clinically meaningful. For example, treatment with SCS was associated with a 43% relative risk reduction in MACE (0.57 [95% CI 0.49–0.67]) and a 51% reduction in all-cause mortality (0.49 [95% CI 0.39–0.62]) over 3 years compared with combination pharmacotherapy. These effect sizes are substantial and comparable to, or greater than, those seen with established cardiometabolic interventions in high-risk populations. Similarly, the 81% reduction in below-knee amputation risk (0.19 [95% CI 0.08–0.46]) is of major clinical relevance given the high morbidity, mortality, and healthcare costs associated with diabetic limb loss. The narrow confidence intervals for most outcomes provide reassurance regarding the precision of these estimates, while the large E-values for key outcomes (e.g., E-value 10.0 for amputation) suggest that the findings are unlikely to be explained by unmeasured confounding alone. Taken together, these measures support the interpretation that SCS offers clinically important benefits beyond pain relief, potentially influencing long-term cardiovascular, limb, and survival outcomes in patients with refractory PDN.

Our results carry clinical relevance. Patients with PDN contribute less to the workforce due to disabling pain intensity, whilst also requiring additional healthcare resources and direct medical costs which add to the economic burden of the disease.^42^ Compared to patients with diabetes without PDN, those with PDN carry a four-fold increased cost to the healthcare system, which increases further in severe PDN.^43^ This is in part driven by prescription of analgesia for neuropathic pain^44^, which is challenging given that around 50% of patients have refractory symptoms despite pharmacotherapy,^10^ largely through burdensome side effects. In total, approximately 77% of patients prescribed pregabalin for PDN will discontinue treatment within 1 year due to limited efficacy or side effects.^11^ To date, only one study has analysed the cost-effectiveness of SCS in PDN and concluded that it was not cost-effective in the short-term.^43^ However, cost-effectiveness can be improved (through two methods: **i)** strict patient selection criteria by screening out patients with non-neuropathic (typically widespread and nociplastic) pain and psychological morbidity, and **ii)** performing trial stimulation and implantation at the same visit^45^), whilst the beneficial cost-effectiveness of SCS will likely not transpire until several years after implantation. SCS involves high upfront costs due to device implantation and monitoring, but the potential long-term benefits demonstrated in our study, as well as reduced medication burden and health care utilisation, may make it cost-effective over time. Further, SCS is explanted in 8–15% of cases, whilst the most serious adverse event (implant site infection) occurs in <1% of cases,^46^ making these systems valid long-term options for patients with refractory PDN. Despite this, only 2.2% of our cohort received SCS, which is in keeping with a UK study whereby only 1% of eligible patients with neuropathic pain had a SCS implanted. The low SCS implant rate over the years may be explained by a lack of awareness of current guidelines and consequent low referral rates for intervention through primary and secondary care.

There are limitations to be acknowledged. These are real-world data and therefore do not provide randomised or controlled comparisons. Second, in data extracted from EMRs in an administrative database, there is potential for a lack of data completeness, and patients may move outside of health care organisations where access to EMRs is not granted. TriNetX is also limited by only including HCOs that are registered to share their EHRs. These types of HCOs are often larger HCOs, which may introduce selection bias. Third, residual bias confounding remains, despite PSM for confounding variables. Variables such as precise alcohol consumption, physical activity, and years lived with diabetes (a risk factor for CVD) are poorly coded using ICD-10 codes, and it should be acknowledged that TriNetX patients largely receive health care in the United States of America using an insurance-based system. The TriNetX database uses EMRs rather than insurance claim data, and therefore information on the type of insurance, and access to healthcare, is limited. To address residual confounding, we present *E*-values from quantitative bias analysis to help readers interpret the strength of our results and reduce the risk of potential unidentified residual confounding. While the large E-values for several key outcomes (e.g., MACE, all-cause mortality, amputation) suggest that substantial unmeasured confounding would be required to fully explain these associations, we acknowledge that for some outcomes, such as staphylococcus aureus infection (upper CI E-value 1.46), the E-values are relatively small. This indicates that even modest unmeasured confounding could attenuate these associations towards the null. Therefore, the results for these outcomes should be interpreted with caution, and confirmation in prospective, controlled studies is required. We have also performed stratified analysis using patients from the USA only to help mitigate the effects of international differences in health care provision. Furthermore, given that outcomes were obtained using ICD-10 codes, TriNetX cannot provide granular information on end points which have traditionally been used in PDN trials such as subjective pain measures, other measures of neuropathy or ischemia, medication burden and quality of life scores. Moreover, we cannot provide an accurate reason for explantation of SCS, nor can we provide an accurate implant success rate. The population studied was also predominantly Caucasian and therefore the results cannot be generalised to all ethnicities. Additionally, TriNetX coding does not allow us to distinguish between low- and HF-SCS. It is likely that not all the patients in this current analysis were treated with HF-SCS, and therefore the associations may vary depending on the type of SCS. Finally, we must acknowledge the innate selection bias in hazard ratios as reported by Hernan; individuals who survive to later periods may differ systematically between treatment groups, not due to the treatment effect but because of inherent differences in susceptibility. Consequently, period-specific hazard ratios might reflect these differences rather than the true effect of the treatment.

There was no formal power calculation performed pre-analysis. This study, with a total of 6210 patients (3105 per treatment arm), represents the largest investigation to date assessing the efficacy of spinal cord stimulation (SCS) in painful diabetic neuropathy (PDN). The primary outcome was evaluated using time-to-event analysis, yielding a hazard ratio of 0.57 with a 95% confidence interval ranging from 0.49 to 0.67. The relatively narrow width of this confidence interval (0.18) reflects a high degree of precision in estimating the treatment effect. Given the large sample size and the precision of the effect estimate, the study was sufficiently powered to detect clinically meaningful differences in treatment efficacy. The narrow confidence interval provides strong evidence that the observed hazard ratio is robust, and the uncertainty around this estimate is limited. Therefore, the sample size is fully justified both based on power and the precision of the estimated treatment effect.

In conclusion, we have demonstrated the impact of SCS on a variety of important long-term health outcomes when treating painful diabetic neuropathy including associated significant risk reductions in major adverse cardiovascular events, all-cause mortality, amputation, suicide, and infection. These real-world data results underline the myriad of clinical effects of SCS and provide justification for including these outcomes in spinal cord stimulation registers for future research.

## Contributors

AEH contributed to the generation of the results and analysis using the TriNetX platform and took the lead on writing the manuscript. DRR and MA assisted with reviewing and editing the manuscript. GH facilitated access to the TriNetX platform, assisted in generating the results, analysis, and review of the final manuscript. RAM, ST, BF and DJC provided senior author input in study design and writing of the manuscript. UA provided senior author review of the manuscript, and oversaw the study development, manuscript writing and provided senior author review of the manuscript. UA is also the guarantor of this work and has full access to all the data and takes responsibility for the integrity of the data and the accuracy of the data analysis. All authors read and approved the final version of the manuscript.

## Data sharing statement

The data that support the findings of this study are available from TriNetX, LLC, https://trinetx.com/, but third-party restrictions apply to the availability of these data. The data were used under license for this study with restrictions that do not allow for the data to be redistributed or made publicly available. However, for accredited researchers, the TriNetX data are available for licensing at TriNetX, LLC. Data access may require a data sharing agreement and may incur data access fees. Data used in the generation of this paper was collected from the global TriNetX network and local data at LUHFT were not used.

## Declaration of interests

MA receives a fellowship from the Novo Nordisk UK research foundation and JDRF. DJC has received investigator-initiated grants from Astra Zeneca and Novo Nordisk, support for education from Perspectum with any financial remuneration from pharmaceutical company consultation made to the University of Liverpool. GHI is an employee of TriNetX LLC. UA has received honoraria from Procter & Gamble, Viatris, Grunenthal and Sanofi for educational meetings and funding for attendance to an educational meeting from Diiachi Sankyo and Sanofi. UA has also received investigator-led funding by Procter & Gamble and is a council member of the Royal Society of Medicine's Vascular, Lipid & Metabolic Medicine Section. RAM has received honoraria from Novo Nordisk, Eli Lilly, Sanofi, Procter & Gamble and Viatris for educational meetings and funding for investigator-initiated studies from Procter & Gamble. ST has received honoraria from Worwag Pharma, Novo Nordisk, Merk, Eva Pharma, Hikma, AstraZeneca, Nevro, P&G Health, Viatris, Berlin-Chemie, Grunenthal, Bayer, NeuroPN, Worwag Pharma, Angelini, Merz (2024), AstraZeneca, NeuroPN, Medtronics andConfotherapeutics. BF is currently the CI for the NIHR HTA trial (133,599) using non-invasive neuromodulation for PDN. GYHL is the co-principal investigator of the AFFIRMO project on multimorbidity in AF, which has received funding from the European Union's Horizon 2020 research and innovation programme under grant agreement No 899871. He is also a consultant and speaker for BMS/Pfizer, Boehringer Ingelheim, Daiichi-Sankyo, Anthos; no fees are received personally. Finally, he is the National Institute for Health and Care Research Senior Investigator, and Chairperson of the Board of the ESC Council on Stroke. All other authors declare that there are no financial relationships or activities that might bias, or be perceived to bias, their contribution to this manuscript.
