# Supplementary material for: Spinal cord stimulation for the treatment of painful diabetic neuropathy and risk of major adverse cardiovascular events, mortality, amputation, infection and suicide: a retrospective cohort study
Source: eClinicalMedicine. 2025 Sep 26;89:103489. doi: 10.1016/j.eclinm.2025.103489 (PMC12508579; doi:10.1016/j.eclinm.2025.103489)
Supplement: Supplementary Tables [file mmc1.docx]

**Supplementary material**

| **Variable** | **ICD-10 or CPT code** |
| --- | --- |
| ***Inclusion criteria*** | |
| Type 1 diabetes | E10 |
| Type 2 diabetes | E11 |
| Spinal cord stimulation | 63685 |
| ***Exclusion criteria*** | |
| Epilepsy | G40 |
| Cerebrovascular accident | I60-I69 |
| Cancer | C00-D49 |
| Chemotherapy-induced peripheral neuropathy | G62.0 |
| Fibromyalgia | M79.7 |
| Spinal cord injury | S14, S24, S34 |

**Supplementary table 1.** Definitions used for inclusion and exclusion criteria. CPT, current procedural term.

| **Variable** | **ICD-10 or CPT code** |
| --- | --- |
| Smoking | F17 |
| Alcohol-use disorder | F10 |
| Cocaine use | F14.1 |
| Socioeconomic status | Z55-Z65 |
| Hypertension | I10-I1A |
| Dyslipidaemia | E78 |
| Ischaemic heart disease | I20-I25 |
| Peripheral vascular disease | I73 |
| Heart failure | I50 |
| Disorders of vitamin and mineral deficiency | E50-E64 |
| Sleep disorders | G47 |
| Migraine | G43 |
| Traumatic brain injury | S00-S09 |
| Depression | F32 |
| Other neuropathies of the peripheral nervous system | G60-G65 |
| Movement disorders | G25 |
| Parkinson’s disease | G20 |

**Supplementary table 2.** Definitions used for propensity score matching variables.

| **Variable** | **ICD-10 or CPT code** |
| --- | --- |
| **Major adverse cardiovascular events** | |
| Ischaemic heart disease | I20-I25 |
| Cerebrovascular accident | I60-I69 |
| Heart failure | I50 |
| Sudden cardiac death | I46.1 |
| **All-cause mortality** | |
| Mortality | Deceased |
| **Below knee amputation** | |
| Tibia and fibula | 1005298 |
| Foot and toes | 1005524 |
| **Suicide** | |
| Suicidal ideation | R45.851 |
| Suicide attempt | T14.91 |
| **Staphylococcus aureus infection** | |
| Infection | B95.6, B95.61, B95.8, A41.0, A41.01, A41.02, A49.01, A49.02, |
| **Major adverse kidney events** | |
| End stage renal failure | N18.6 |
| Dialysis | 1012740 |
| **Diabetes-related ophthalmic disease** | |
| Type 1 diabetes mellitus with ophthalmic complications | E10.3 |
| Type 2 diabetes mellitus with ophthalmic complications | E11.3 |
| **Hospitalisation** | |
| Hospital Inpatient Services | 1013659 |
| **Explant** | |
| Revision or removal of an implanted spinal neurostimulator | 63688 |

**Supplementary table 3.** Definitions used for outcomes.

| **Stratified analysis** | **Hazard ratio [95% confidence interval]** |
| --- | --- |
| ***Major adverse cardiovascular events*** | |
| ***Sex*** | |
| Male | ***0.54 [0.43, 0.67]*** |
| Female | ***0.58 [0.45, 0.73]*** |
| ***Ethnicity*** | |
| White ethnicity | ***0.56 [0.46, 0.67]*** |
| Non-white ethnicity | ***0.67 [0.49, 0.91]*** |
| ***Age*** | |
| Older | ***0.60 [0.51, 0.71]*** |
| Younger | ***0.50 [0.34, 0.73]*** |
| ***Discontinuation of medical therapy*** | |
| Treatment discontinued | ***0.37 [0.27, 0.51]*** |
| ***Geographical location*** | |
| USA | ***0.52 [0.45, 0.61]*** |
| ***Mortality*** | |
| ***Sex*** | |
| Male | ***0.44 [0.33, 0.58]*** |
| Female | ***0.34 [0.24, 0.49]*** |
| ***Ethnicity*** | |
| White ethnicity | ***0.40 [0.32, 0.51]*** |
| Non-white ethnicity | ***0.36 [0.22, 0.58]*** |
| ***Age*** | |
| Older | ***0.47 [0.37, 0.59]*** |
| Younger | ***0.25 [0.13, 0.50]*** |
| ***Discontinuation of medical therapy*** | |
| Treatment discontinued | ***0.52 [0.35, 0.75]*** |
| ***Geographical location*** | |
| USA | ***0.41 [0.33, 0.51]*** |
| ***Below knee amputation*** | |
| ***Sex*** | |
| Male | ***0.27 [0.09. 0.80]*** |
| Female | 0.34 [0.07, 1.69] |
| ***Ethnicity*** | |
| White ethnicity | ***0.28 [0.10, 0.75]*** |
| Non-white ethnicity | 0.21 [0.03, 1.83] |
| ***Age*** | |
| Older | ***0.18 [0.05, 0.60]*** |
| Younger | ***0.25 [0.07, 0.90]*** |
| ***Discontinuation of medical therapy*** | |
| Treatment discontinued | ***0.11 [0.01, 0.81]*** |
| ***Geographical location*** | |
| USA | ***0.20 [0.08, 0.51]*** |
| ***Suicide*** | |
| ***Sex*** | |
| Male | ***0.37 [0.30. 0.68]*** |
| Female | ***0.37 [0.17, 0.84]*** |
| ***Ethnicity*** | |
| White ethnicity | ***0.30 [0.18, 0.52]*** |
| Non-white ethnicity | 0.35 [0.11, 1.09] |
| ***Age*** | |
| Older | ***0.50 [0.27, 0.93]*** |
| Younger | ***0.25 [0.12, 0.51]*** |
| ***Discontinuation of medical therapy*** | |
| Treatment discontinued | ***0.24 [0.08, 0.71]*** |
| ***Geographical location*** | |
| USA | ***0.41 [0.25, 0.67]*** |
| ***Staphylococcus aureus infection*** | |
| ***Sex*** | |
| Male | 0.94 [0.64, 1.39] |
| Female | ***0.54 [0.34, 0.88]*** |
| ***Ethnicity*** | |
| White ethnicity | ***0.62 [0.45, 0.87]*** |
| Non-white ethnicity | 0.83 [0.47, 1.47] |
| ***Age*** | |
| Older | ***0.50 [0.36, 0.69]*** |
| Younger | 0.94 [0.58, 1.51] |
| ***Discontinuation of medical therapy*** | |
| Treatment discontinued | ***0.36 [0.18, 0.71]*** |
| ***Geographical location*** | |
| USA | ***0.63 [0.48, 0.84]*** |
| ***Major adverse kidney events*** | |
| ***Sex*** | |
| Male | 0.55 [0.26, 1.15] |
| Female | 0.65 [0.25, 1.68] |
| ***Ethnicity*** | |
| White ethnicity | ***0.44 [0.24, 0.81]*** |
| Non-white ethnicity | ***0.33 [0.11, 0.99]*** |
| ***Age*** | |
| Older | 0.59 [0.33, 1.04] |
| Younger | NA |
| ***Discontinuation of medical therapy*** | |
| Treatment discontinued | ***0.30 [0.11, 0.78]*** |
| ***Geographical location*** | |
| USA | ***0.35 [0.21, 0.58]*** |
| ***Diabetes-related ophthalmic disease*** | |
| ***Sex*** | |
| Male | ***0.38 [0.22, 0.64]*** |
| Female | ***0.43 [0.25, 0.74]*** |
| ***Ethnicity*** | |
| White ethnicity | ***0.43 [0.28, 0.67]*** |
| Non-white ethnicity | 0.56 [0.28, 1.14] |
| ***Age*** | |
| Older | ***0.43 [0.28, 0.65]*** |
| Younger | ***0.38 [0.18, 0.81]*** |
| ***Discontinuation of medical therapy*** | |
| Treatment discontinued | ***0.22 [0.10, 0.48]*** |
| ***Geographical location*** | |
| USA | ***0.33 [0.22, 0.47]*** |
| ***Hospitalisation*** | |
| ***Sex*** | |
| Male | ***0.63 [0.54, 0.74]*** |
| Female | ***0.54 [0.45, 0.64]*** |
| ***Ethnicity*** | |
| White ethnicity | ***0.56 [0.49, 0.64]*** |
| Non-white ethnicity | ***0.78 [0.63, 0.96]*** |
| ***Age*** | |
| Older | ***0.60 [0.53, 0.67]*** |
| Younger | ***0.60 [0.48, 0.75]*** |
| ***Discontinuation of medical therapy*** | |
| Treatment discontinued | ***0.28 [0.22, 0.36]*** |
| ***Geographical location*** | |
| USA | ***0.55 [0.49, 0.61]*** |

**Supplementary table 4.** Stratified analyses by sex, age, and ethnicity.
